# The perception of surgical valve disease patients on quality of life improvement through the care line model: a longitudinal study

**DOI:** 10.3389/fcvm.2025.1489309

**Published:** 2026-01-23

**Authors:** Sirlei Pereira Nunes, Vitor Emer Egypto Rosa, Danielle Misumi Watanabe, Bellkiss Wilma Romano, Flávio Tarasoutchi

**Affiliations:** Instituto do Coração (InCor), Hospital das Clínicas HCFMUSP, Faculdade de Medicina, Universidade de São Paulo, São Paulo, Brazil

**Keywords:** cardiac surgery, care line, quality of life, value-based health care, valvular heart diseases

## Abstract

**Objectives:**

To evaluate the improvement in the quality of life of surgical cardiac valve disease patients based on their perceptions at three distinct points: preoperative, immediate postoperative, and late postoperative.

**Background:**

Quality of life has been increasingly recognized as a central outcome in cardiovascular care, especially in valvular diseases. Despite the extensive international literature on postoperative recovery and favorable clinical outcomes, few studies have examined quality of life from the patient's perspective within a structured care pathway model, particularly in developing countries such as Brazil. This study contributes by assessing quality of life longitudinally and from the patient's perspective, within an interdisciplinary Care Line Model implemented at a high-complexity cardiovascular center.

**Methods:**

This retrospective, observational longitudinal study included patients with significant valvular disease undergoing surgery. These patients were assessed at three time points by the psychology team: preoperative, immediate postoperative (after discharge from the Intensive Care Unit and before hospital discharge), and late postoperative (6 months after hospital discharge). Quality of life was measured from the patients' perspective using two instruments: SF-36 and EQ-5D, as part of the surgical valve disease care model implemented at the institution.

**Results:**

Patients reported significant improvements in quality of life after surgery. The EQ-5D and EQ-VAS scores increased substantially in the late postoperative period compared to preoperative values. SF-36 domains, particularly functional capacity, vitality, pain, general health, and mental health, showed robust improvement. All analyses were based strictly on comparisons between assessment points; no assumptions of linear postoperative improvement were made.

**Conclusion:**

Valve surgery is associated with meaningful improvements in patients’ perceived quality of life, especially regarding mobility, pain/discomfort, self-care, and emotional and social functioning. These findings reinforce the relevance of multidisciplinary and longitudinal follow-up and demonstrate the potential contribution of structured care pathways, such as the Surgical Valve Disease Care Line, to enhance recovery and patient-centered outcomes.

## 1 Introduction

Over the past decade, numerous healthcare institutions worldwide have increasingly incorporated patient-centered care principles and value-based health care models into clinical practice. Patient experience has become a core pillar in health service evaluation, requiring the development of innovative, structured, and patient-focused care strategies. The implementation of care lines has emerged as a promising approach, aiming to coordinate multidisciplinary practices and unify therapeutic pathways according to the needs of specific patient populations (1–5).

A patient-centered approach emphasizes the value patients attribute to their own health and quality of life. In this perspective, care becomes a shared responsibility across the entire multidisciplinary team, currently known as the Heart Team, which includes cardiologists, surgeons, anesthetists, nurses, nutritionists, physiotherapists, psychologists, and other professionals. This model is endorsed globally as an effective strategy for improving outcomes and aligning care with patients' priorities (6, 7).

Quality of life (QoL) has gained increasing importance in cardiovascular research, particularly in chronic conditions such as valvular heart disease, where functional deterioration, symptom progression, and psychosocial burden significantly impact daily life. Understanding QoL as a personal, subjective, and non-transferable construct allows clinicians and managers to evaluate care beyond clinical indicators (8–11).

Although the literature demonstrates consistent improvements in QoL following clinical and surgical treatments for valvular disease, studies vary in methodological rigor, measurement instruments, and follow-up strategies. Moreover, there is still limited evidence examining QoL specifically from the patient's perspective within structured care pathways, particularly in developing countries, where care models, access, and socioeconomic determinants differ substantially from those in developed nations. This gap has been highlighted by recent publications emphasizing the need for conceptual and methodological refinement in the assessment and reporting of quality of life (11–14).

Additionally, while international literature on postoperative QoL after valve surgery and TAVI has grown substantially, few studies contextualize these outcomes within multidisciplinary, longitudinal, and institutionalized care models. The Care Line Model implemented at our institution represents an innovation by systematically integrating psychological assessment into the perioperative care of valve patients, allowing for repeated QoL measurement at critical recovery stages (15, 16).

Socioeconomic and demographic factors, such as educational level, occupational demands, and gender, are also known to influence QoL in cardiovascular populations, yet these dimensions remain underexplored in surgical valve cohorts. Recent studies reinforce that social determinants significantly shape health-related quality of life, recovery expectations, and long-term adaptation after heart surgery (17, 18).

Taking these factors together, this study aims to contribute to the literature by evaluating QoL longitudinally at three perioperative time points, using validated patient-reported outcome measures (SF-36 and EQ-5D), embedded within a structured Surgical Valve Disease Care Line. This model enables standardized follow-up, interdisciplinary collaboration, and improved understanding of the patient's subjective recovery trajectory.

Therefore, the objective of the present study was to evaluate the improvement in quality of life from the perspective of surgical cardiac valve disease patients at three distinct time points: preoperative, immediate postoperative (after discharge from the Intensive Care Unit and before hospital discharge), and late postoperative (6 months after hospital discharge), as assessed through the Surgical Valve Disease Care Line.

## 2 Methods

### 2.1 Study population and care protocol

This retrospective observational longitudinal study analyzed data sequentially extracted from a control spreadsheet created and archived by the Psychology Service of the Heart Institute, from March 2018 to March 2020. The psychological evaluations were conducted at three points: preoperative in the outpatient clinic, immediate postoperative (after discharge from the Intensive Care Unit and before hospital discharge), and late postoperative (6 months after hospital discharge).

**Figure 1:**
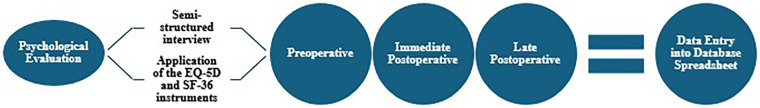
Flowchart of the psycholotical care routine m the care protocol of the surgical valve disease care line. Description: psychological evaluation using the semi-structured interview technique and the application of the SF-36 and EQ-SD instruments to assess and measure the quality of life from the perception of the surgical valve disease patient at three moments: preoperative, immediate postoperative (after discharge from the intensive care unit and before hospital discharge), and late postoperative (6 months from hospital discharge). After the evaluation and corrections of the applied instruments, the data were entered into the database spreadsheet.

The assessments were conducted within the Surgical Valve Disease Care Line (Figure 1), a structured clinical pathway implemented by the institution to standardize preoperative and postoperative follow-up.

The Care Line Model is a structured, multidisciplinary protocol that integrates sequential assessments by cardiology, surgery, nursing, rehabilitation teams, and psychology. Unlike traditional fragmented follow-up, this model ensures continuous, interdisciplinary monitoring of patients from preoperative evaluation to long-term postoperative recovery. It also standardizes the timing and content of psychological assessments, enabling consistent measurement of patient-reported outcomes over time.

The team involved includes clinical cardiologists, surgeons, anesthetists, dentists, nurses, pharmacists, nutritionists, social workers, physiotherapists, psychologists, and other professionals. Patients were eligible if they were indicated for valve intervention, regardless of waiting time, number of previous surgeries, or valve prosthesis type (biological or mechanical), were between 18 and 65 years old, had any level of education, and were followed by the Heart Institute outpatient clinic after intervention.

Exclusion criteria were:

1. Patients are not evaluated by psychology at all three points.
2. Patients referred to external outpatient follow-up or who discontinued treatment.
3. Patients presenting cognitive deficits (memory, attention, language, reasoning, concentration) or psychiatric diagnoses (schizophrenia, bipolar disorder, personality disorders) that compromised QoL instrument validity. The final sample consisted of 133 participants.

The study was approved by the ethics committee, which waived the requirement for informed consent in accordance with Resolution 466/12.

### 2.2 Instruments

#### 2.2.1 Database

Refers to an Excel control spreadsheet created and archived by the Heart Institute psychology service. The sociodemographic and clinical data stored in this spreadsheet were extracted from the form entitled “Personal Data of the EQ-5D Instrument (Supplementary Material)” and the institution's electronic medical record S3: age, sex, education level, occupational/activity status, smoking, alcohol/drug use, disease etiology, psychiatric and psychological history, use of psychotropic medications, performed valve surgical procedure, type of prosthesis (biological, mechanical, or no prosthesis replacement).

#### 2.2.2 Quality of life instruments

Both instruments used are validated for use in Brazil and widely adopted in cardiovascular outcomes research.

### 2.3 SF-36 (Brazilian version)

A 36-item self-report questionnaire assessing eight domains of health-related quality of life. Scores range from 0 to 100, with higher scores indicating better perceived health (19) (Supplementary Material).

### 2.4 EQ-5D (Brazilian version)

Assesses five dimensions (mobility, self-care, usual activities, pain/discomfort, anxiety/depression) using three-level responses, plus a visual analogue scale (0–100) (20) (Supplementary Material).

### 2.5 Statistical analysis

In the descriptive analysis, data were expressed as absolute and relative frequencies for categorical variables and as mean and standard deviation for numerical variables. The probability distribution models adopted in the analyses were defined according to data adherence based on the Quasi-Likelihood under Independence Model Criterion (QIC). According to this criterion, a lower QIC value indicates better data adherence to the model (21).

The evolution of quality of life after valve surgical intervention was assessed using Generalized Estimating Equations (GEE) for the SF-36 scale domains. Since the SF-36 consists of discrete numerical variables, normal and Poisson distribution models were compared. The Poisson distribution showed the lowest QIC values in all models performed (Table 1).

**Table 1:** QIC values for regression models of the SF-36 instrument domains.

Regression model	Social functioning		Functional capacity		Pain		General health		Physical aspects		Role-emotional		Mental health		Vitality	
	Normal	Poisson	Normal	Poisson	Normal	Poisson	Normal	Poisson	Normal	Poisson	Normal	Poisson	Normal	Poisson	Normal	Poisson
Time	4,010,75.98	−164,547.65	339,164.76	−119,354.52	353,107.62	−148,921.54	249,587.9	−169,049.45	702,210.42	−168,131.78	573,492.71	−104,960.24	235,335.61	−172,215.10	95,667.51	−154,240.39
Sex	432,987.98	−164,063.31	410,024.47	−118,035.07	353,662.70	−148,914.59	260,530.46	−168,878.17	737,146.74	−167,611.77	759,856.62	−101,026.63	226,330.36	−172,346.35	99,776.94	−154,176.78
Age group	440,262.84	−163,953.07	424,880.36	−117,751.93	360,801.80	−148,798.56	267,500.52	−168,775.20	747,347.93	−167,461.07	765,971.66	−100,894.11	240,629.88	−172,138.64	103,299.59	−154,120.31
Education level	425,609.64	−161,659.92	406,038.91	−116,806.07	348,479.92	−146,578.67	261,911.82	−166,499.81	736,147.72	−164,070.13	730,454.89	−99,708.37	235,413.88	−169,721.11	100,711.85	−151,361.23
Professional activity	426,444.34	−164,155.36	409,537.29	−118,042.12	347,856.91	−149,003.28	261,834.48	−168,853.13	736,810.68	−167,603.96	750,640.99	−101,232.70	238,356.14	−172,163.71	102,773.50	−154,122.17
Smoking	419,553.24	−164,303.54	423,675.17	−117,774.52	352,852.12	−148,935.52	268,882.66	−168,752.26	723,015.06	−167,871.45	761,164.26	−101,005.82	227,048.19	−172,352.25	98,869.03	−154,191.88
Alcohol and drugs	440,490.87	−163,949.18	424,875.02	−117,752.67	357,277.67	−148,861.42	269,300.68	−168,748.69	750,286.23	−167,416.91	764,967.74	−100,915.74	236,829.99	−172,198.87	101,668.36	−154,146.35
Psychiatric and psychological history	427,691.30	−164,157.38	419,956.21	−117,853.31	348,829.55	−149,007.94	268,792.76	−168,755.76	730,881.46	−167,731.49	751,407.22	−101,246.52	233,740.11	−172,243.68	100,795.47	−154,161.33
Psychotropic medications	421,040.54	−164,267.84	402,467.27	−118,233.50	334,714.57	−149,265.71	264,889.82	−168,816.36	724,123.48	−167,842.48	745,044.04	−101,404.71	226,562.10	−172,357.13	99,449.55	−154,183.63
Etiology	439,905.69	−163,953.50	414,972.61	−117,931.08	349,823.41	−148,975.57	263,316.71	−168,832.13	734,781.45	−167,659.53	748,568.43	−101,271.93	237,988.98	−172,172.99	102,951.51	−154,120.11
Procedure performed	440,026.52	−163,954.71	425,122.32	−117,745.39	348,865.75	−148,998.29	268,106.58	−168,764.20	740,991.07	−167,557.45	765,012.29	−100,913.41	240,163.30	−172,143.51	103,206.91	−154,119.86
Type of valve	440,385.99	−163,949.24	425,422.71	−117,739.46	358,586.02	−148,832.61	269,327.12	−168,745.93	746,166.63	−167,475.53	763,254.86	−100,950.62	239,723.15	−172,150.54	102,657.15	−154,129.71
Multivariate	333,658.94	−163,107.43	280,212.05	−119,207.42	289,401.54	−149,971.50	217,902.79	−169,507.89	627,404.47	−169,305.85	505,293.62	−104,560.88	188,098.26	−172,922.52	84,179.71	−154,417.54

For the EQ-5D scale and its dimensions, which are continuous variables, normal and Gamma distribution models were compared. Since the Gamma distribution only allows positive values greater than zero and the EQ-5D scale and its dimensions presented negative or zero values, the EQ-5D scale and its dimensions were initially transformed for model comparison (Table 2). This transformation involved adding a constant to all variable values, defined as one plus the minimum value of the variable. The normal distribution presented the lowest QIC values in all analyzed models (Table 2). As the normal distribution model demonstrated better adherence, the analyses were conducted with the real (non-transformed) variables.

**Table 2:** QIC values for regression models of the total score and dimensions of the EQ-5D instrument and the visual analog scale (EQVAS).

Regression model	EQ5D		Anxiety/Depression		Usual activities		Self-Care		Pain/Discomfort		Mobility		EQVAS	
	Normal	Gamma	Normal	Gamma	Normal	Gamma	Normal	Gamma	Normal	Gamma	Normal	Gamma	Normal	Poisson
Time	20.73	1,147.02	7.62	837.21	6.32	811.22	6.87	812.74	9.46	859.30	20.73	1,147.02	180,648.33	−176,294.73
Sex	19.39	1,145.4	5.44	835.05	4.29	809.2	4.66	810.55	7.12	856.99	19.39	1,145.40	203,607.42	−175,950.39
Age group	20.02	1,145.97	5.54	835.12	4.05	808.95	4.84	810.72	7.09	856.94	20.02	1,145.97	207,629.94	−175,892.48
Education level	30.67	1,140.26	14.41	831.37	16.70	809.15	14.40	807.65	20.05	856.57	30.67	1,140.26	203,882.22	−172,804.21
Professional activity	27.38	1,153.17	11.46	841.11	11.17	816.08	10.43	816.33	13.89	863.74	27.38	1,153.17	204,492.94	−175,931.03
Smoking	21.56	1,147.7	8.57	838.06	7.01	811.88	6.96	812.80	10.09	859.83	21.56	1,147.70	204,316.29	−175,939.28
Alcohol and drugs	19.98	1,145.98	6.13	835.68	5.42	810.3	5.02	810.86	7.54	857.35	19.98	1,145.98	206,303.01	−175,911.24
Psychiatric and psychological history	20.19	1,146.38	6.40	835.93	4.82	809.7	5.01	810.86	7.96	857.66	20.19	1,146.38	205,410.78	−175,924.64
Psychotropic medications	19.48	1,145.91	6.10	835.64	4.76	809.63	5.13	810.96	8.02	857.7	19.48	1,145.91	203,869.1	−175,947.52
Etiology	25.66	1,151.53	10.41	840.03	10.87	815.77	11.02	816.90	11.91	861.78	25.66	1,151.53	202,503.99	−175,962.05
Procedure performed	22.06	1,148.02	7.37	836.98	6.09	810.99	6.86	812.74	9.73	859.54	22.06	1,148.02	207,726.39	−175,888.72
Type of valve	22.12	1,148.07	7.38	837.00	5.79	810.69	7.01	812.88	9.45	859.27	22.12	1,148.07	207,582.08	−175,891.25
Multivariate	30.48	162,581.92	31.42	860.84	10.98	815.86	27.83	821.05	13.55	863.26	22.95	833.00	162,581.91	−176,548.50

The adjusted (multivariate) GEE model was applied to variables that were statistically significant (*p* < 0.05). Tukey's multiple comparisons were used when necessary. In all analyses, the significance level was set at 5%, and the analyses were conducted using R statistical computing software version 4.0.5 (R Foundation for Statistical Computing, Vienna, Austria) (22).

## 3 Results

The sociodemographic and clinical characteristics of the study sample are described in Table 3. Among the 133 patients analyzed, the average age was 50.8 years. Gender distribution showed that 90 patients (67.7%) were female. Regarding education, 55 patients (41.4%) reported having a low educational level, indicating incomplete elementary education. The predominant occupation in the sample was homemaking, representing 36 patients (27.1%). Additionally, 15 patients (11.3%) were smokers, 22 patients (16.5%) had psychiatric and psychological histories, and 25 patients (18.8%) were on psychotropic medications. Five patients (3.8%) reported the use of alcohol and illicit drugs. Concerning etiology, rheumatic fever was the most prevalent, affecting 73 patients (54.9%). Mitral valve replacement was the most common surgical intervention, occurring in 58 patients (43.6%), with a biological valve chosen by 95 patients (71.4%).

**Table 3:** Sociodemographic and clinical characteristics of the population represented in the present study of patients who underwent valvular heart surgery from March 2018 to March 2020.

Variable	Description	*N* = 133
Sex	Female	90 (67.7%)
Age, years		50.8 ± 10.3
Education level	Incomplete elementary	55 (41.4%)
	Elementary	20 (15%)
	High School	31 (23.3%)
	Higher education	10 (23.3%)
Professional activity	Retired	19 (14.3%)
	Self-employed	11 (8.3%)
	Homemaker	36 (27.1%)
	Employed	35 (26.3%)
	Others	30 (22.6%)
Smoking	Former smoker	44 (33.1%)
	Smoker	15 (11.3%)
Alcohol and drugs	Yes	5 (3.8%)
Psychiatric and psychological history	Yes	22 (16.5%)
Psychotropic medications	Yes	25 (18.8%)
Etiology	To Be Clarified	31 (23.3%)
	Congenital/Bicuspid	6 (4.5%)
	Degenerative	7 (5.3%)
	Rheumatic fever	73 (54.9%)
	Mitral valve prolapse	16 (12%)
Procedure performed	Others	33 (24.8%)
	Aortic valve replacement	42 (31.6%)
	Mitral valve replacement	58 (43.6%)
Type of valve	Biological	95 (71.4%)
	Mechanical	18 (13.5%)
	There was no prosthesis replacement	20 (15%)

### 3.1 Quality of life perception based on EQ-5D and EQ-VAS

**Figure 2:**
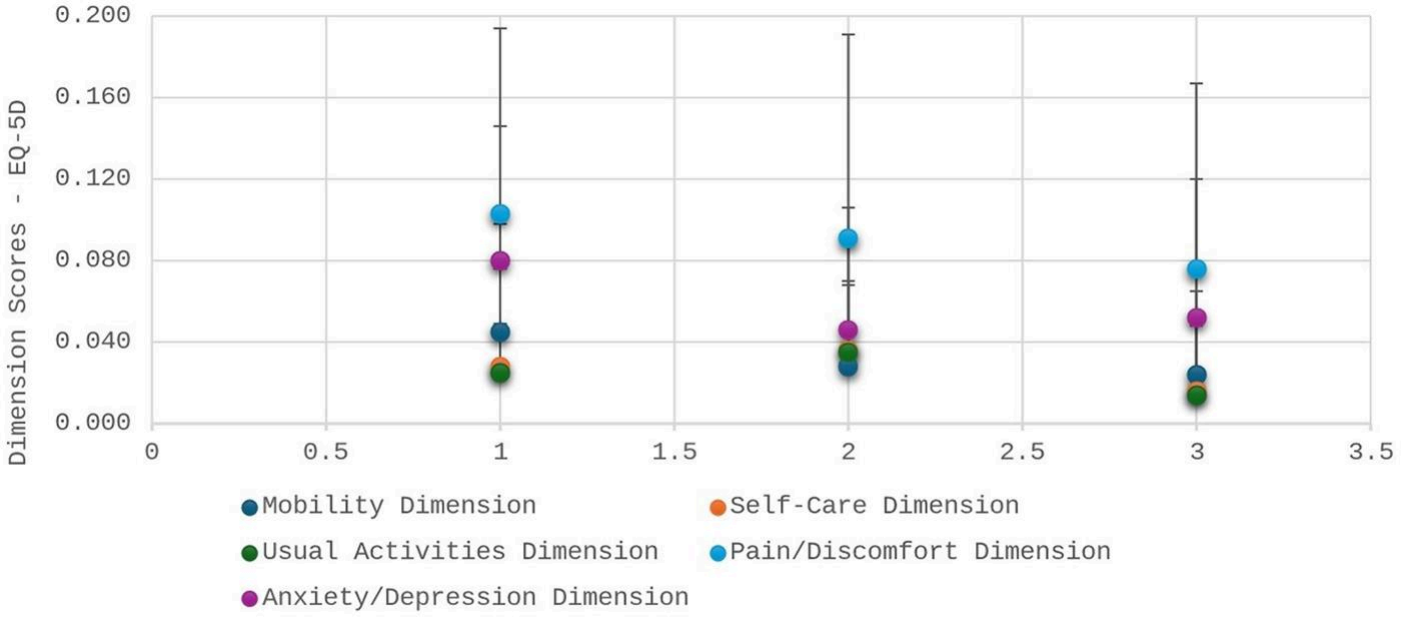
Data expressed as mean and standard deviation of the EQ-5D instrument score. ***Represents *p* < 0.001 compared to preoperative and postoperative evaluations in the ward. The horizontal bar represents the time points of EQ-5D administration: (1) preoperative; (2) immediate postoperative (after ICU discharge and before hospital discharge); and (3) late postoperative (6 months after hospital discharge).

**Figure 3:**
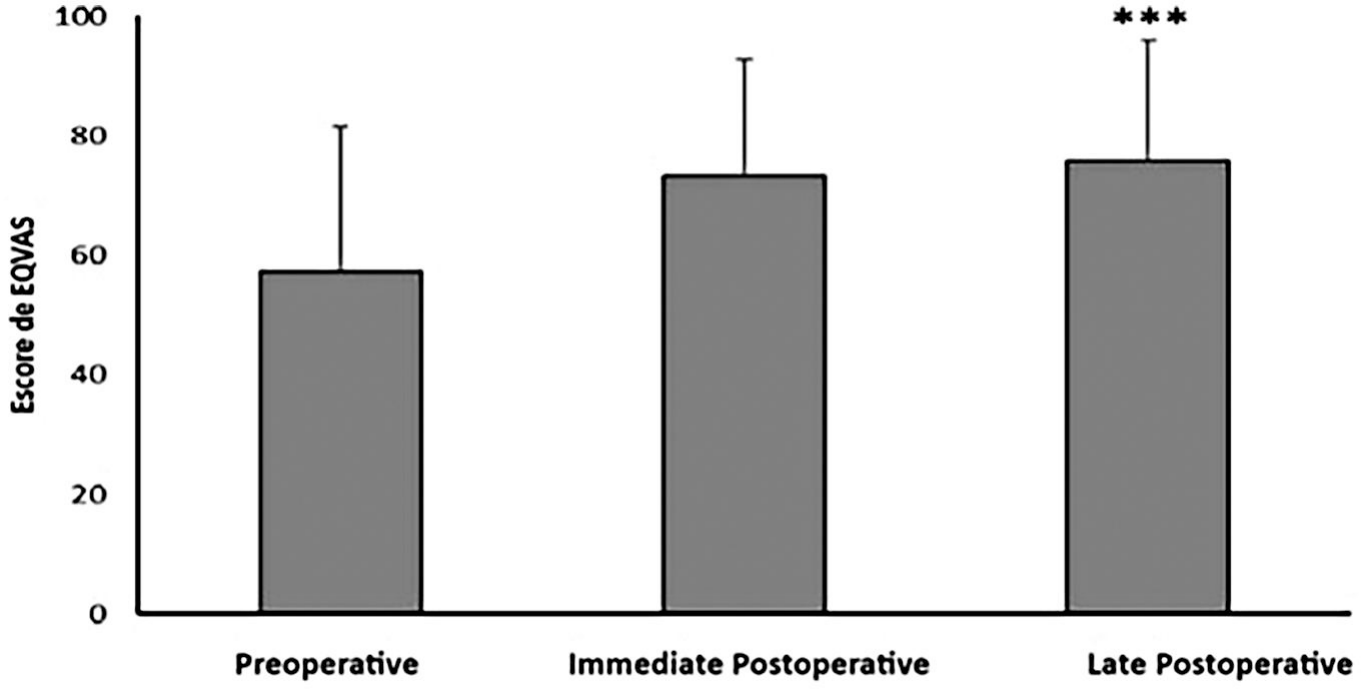
Data expressed as mean and standard deviation of the EQVAS score of the EQ-5D instrument. ***Representa *p* < 0.001 em comparação com a avaliação no pré-operatório.

Patients reported improvements in several dimensions of quality of life after valve surgery, as evidenced by EQ-5D results (Table 4; Figures 2, 3). The total EQ-5D score increased from 0.587 preoperatively to 0.723 in the late postoperative period, indicating a global enhancement in perceived health status.

**Table 4:** Mean and standard deviation of the total score and dimensions of the EQ-5D-BR instrument and the visual analog scale (EQVAS) for patients who underwent valvular surgery at INCOR-HCFMSUP from March 2018 to March 2020.

EQ-5D Instrument/Dimensions	Preoperative	Immediate postoperative	Late postoperative
EQ5D-BR	0.587 (0.178)	0.631 (0.196)	0.723 (0.205)
Mobility	0.045 (0.053)	0.028 (0.042)	0.024 (0.041)
Personal care	0.028 (0.048)	0.036 (0.053)	0.016 (0.037)
Usual activities	0.025 (0.024)	0.035 (0.033)	0.014 (0.034)
Pain/Illness	0.103 (0.091)	0.091 (0.100)	0.076 (0.091)
Anxiety/Depression	0.080 (0.066)	0.046 (0.060)	0.052 (0.068)
EQVAS	57,200 (24,300)	73,400 (19,600)	75,900 (20,100)

Improvements were observed in the following areas:

- Mobility: scores decreased (indicating fewer mobility problems), from 0.045 preoperatively to 0.024 in late postoperative follow-up.
- Self-care: although scores slightly worsened in the immediate postoperative period, they improved to 0.016 at late follow-up.
- Usual activities: showed recovery from 0.025 to 0.014.
- Pain/discomfort: symptoms reduced from 0.103 to 0.076.
- Anxiety/depression: decreased from 0.080 to 0.046 immediately after surgery, with a mild increase to 0.052 at 6 months.

In the EQ-VAS, patients rated their overall health markedly higher in the late postoperative period (75.9) compared to preoperative assessment (57.2).

### 3.2 Quality of life perception based on SF-36

**Figure 4:**
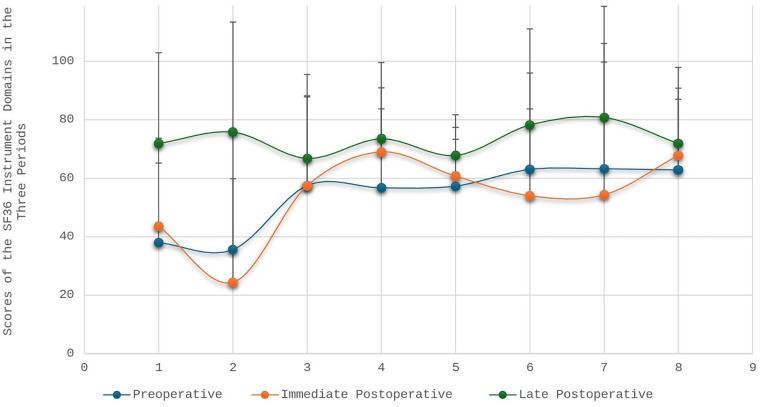
Scores of the 8(eight) domains of the SF36 instrument at the three moments of quality of life assessment by the surgical valvopathy care line. The horizontal bar represents the eight domains of the SF-36 instrument: (1) physical functioning; (2) role limitations due to physical problems; (3) pain; (4) general health; (5) vitality; (6) social functioning; (7) role limitations due to emotional problems; and (8) mental health.

Table 5 and Figure 4 illustrate significant improvements across nearly all SF-36 domains in the late postoperative period.

**Table 5:** Mean and standard deviation of the domain scores of the SF36 instrument for patients who underwent valve surgery at INCOR-HCFMSUP from March 2018 to March 2020.

SF36 Instrument/Domains	Preoperative	Immediate postoperative	Late postoperative
Functional capacity	38.0 (27.2)	43.6 (30.1)	71.9 (31.0)
Physical aspects	35.6 (41.3)	24.4 (35.4)	75.8 (37.6)
Pain	57.5 (30.7)	57.4 (30.4)	66.8 (28.7)
General health	56.7 (27.0)	69.0 (21.9)	73.5 (26.1)
Vitality	57.3 (16.0)	60.7 (16.7)	67.8 (13.9)
Social functioning	63.0 (33.0)	54.0 (29.7)	78.2 (32.9)
Role-emotional	63.2 (42.9)	54.3 (45.4)	80.8 (38.0)
Mental health	62.8 (24.2)	67.8 (23.0)	71.9 (26.0)

Key findings include:

- Physical functioning: increased from 38.0 preoperatively to 71.9 6 months post-discharge.
- Role limitations due to physical problems: initially worsened immediately after surgery (24.4), followed by substantial recovery (75.8).
- Pain: improved from 57.4 to 66.8.
- General health perceptions: increased from 56.7 to 73.5.
- Vitality: improved from 57.3 to 67.8.
- Social functioning: decreased shortly after surgery (54.0) but improved significantly by 6 months (78.2).
- Role-emotional: similar pattern, from 54.3 immediately after surgery to 80.8 in the late postoperative period.
- Mental health: increased steadily from 62.8 to 71.9.

All domain improvements were interpreted as discrete changes between the three observed periods, without attributing linearity or proportional increases over time.

## 4 Discussion

The present study demonstrated significant improvements in quality of life (QoL) among patients undergoing cardiac valve surgery, as assessed at three perioperative time points using the EQ-5D and SF-36 instruments. These findings corroborate the established evidence that surgical intervention improves functional status, symptom burden, and general well-being in valvular heart disease (23, 24). However, this study provides additional contributions by evaluating QoL longitudinally within a structured care pathway and integrating psychological assessment as part of the multidisciplinary follow-up.

Cardiovascular diseases remain the leading cause of morbidity and mortality worldwide, and QoL deterioration is a major determinant of disease burden in chronic heart disease. Surgical intervention is often the only viable treatment for individuals with advanced valvular disease, restoring hemodynamic function and mitigating symptoms. Our findings align with previous literature indicating that patients experience substantial improvements in mobility, pain, emotional status, and functional domains after valve surgery (25–28).

A central contribution of the present study is that, despite the existing literature on postoperative QoL, few studies evaluate these outcomes within a structured, multidisciplinary Care Line Model. The longitudinal and protocolized nature of this model allowed for standardized perioperative assessment, enhancing the reliability of patient-reported outcomes and strengthening the potential for clinical applicability. This study therefore provides evidence that organizational care models may directly influence patient recovery and perceived QoL.

Furthermore, this research emphasizes the subjective dimension of QoL, understanding it as a personal and context-dependent experience, which is especially relevant in low- and middle-income countries where socioeconomic disparities, resource availability, and access to rehabilitation services vary widely. These contextual elements justify the importance of generating local evidence, even when international literature on QoL after valve surgery is extensive.

### 4.1 Quality of life instruments and rationale for their use

Instruments were based on their strong psychometric properties, their wide adoption in cardiovascular outcomes research, and their ability to capture both general and health-specific quality of life domains. Additionally, both instruments allow comparison with international datasets and are validated for use in Brazilian populations. Studies highlight that QoL is a multifaceted construct, and no single instrument fully captures its complexity, hence the relevance of using two complementary scales (29, 30).

The difficulty in defining and operationalizing QoL, highlighted in classical works such as Gill and Feinstein (30) and reaffirmed in more recent analyses (31), underscores the importance of using reliable methodological frameworks when assessing patient trajectories. By applying two validated instruments at three different postoperative stages, this study adds methodological rigor to the understanding of QoL in valvular disease (9, 30–32).

### 4.2 Influence of sociodemographic variables

Sociodemographic characteristics, particularly education, occupation, and gender, play a significant role in shaping QoL outcomes. Evidence from congenital and acquired heart disease indicates that socioeconomic status affects patients' ability to adapt to surgical recovery, access rehabilitation resources, and sustain long-term lifestyle changes. Our sample profile, which included predominantly women, individuals with low educational level, and a significant proportion of homemakers, reflects a sociodemographic configuration that may influence perceptions of recovery and the psychosocial demands of postoperative adjustment. These dimensions warrant further study and should be considered when designing individualized follow-up strategies within care pathways (33).

### 4.3 Comparison with international literature

The present findings are consistent with systematic reviews and clinical studies reporting that valve surgery leads to substantial improvements in functioning, vitality, and symptom burden. Postoperative gains in mobility, self-care, and emotional health observed here parallel gains demonstrated in large observational courts and randomized studies (6, 34–37).

More recent investigations (2021–2025) emphasize early mobilization, functional recovery trajectories, and patient-reported outcomes following cardiac surgery. These studies reinforce the role of structured and interdisciplinary care models in optimizing postoperative QoL. Incorporating these perspectives strengthens the relevance of our findings and situates the present study within contemporary international discussions (38–40).

### 4.4 Contribution of the surgical valve disease care line

The Care Line Model represents an innovative contribution by institutionalizing interdisciplinary follow-up and embedding psychological assessment into routine perioperative care. The structured sequence of appointments ensures that QoL data is collected consistently at clinically meaningful moments, enabling the detection of psychosocial needs that may emerge during recovery. This framework not only improves data quality but also reinforces patient-centered care, aligning with value-based health care principles (41, 42).

Given the global emphasis on patient-centered outcomes and integrated care pathways, our findings support the integration of structured models into cardiovascular surgical programs, particularly in settings where fragmentation of care persists (43, 44).

### 4.5 Clinical implications

All analyses were interpreted as discrete comparisons between the three time periods. The improvements observed at 6 months should not be understood as linear or progressive trends throughout the postoperative period, but rather as cumulative effects of surgery, rehabilitation, and psychosocial adaptation. This approach reinforces the clinical importance of longitudinal rather than continuous assessment models to ensure long-term recovery. Psychosocial support is particularly relevant given the fluctuations observed in anxiety and depression scores, emphasizing that emotional adaptation may not follow the same trajectory as physical recovery. Incorporating psychological assessment into routine postoperative care may facilitate the early identification of vulnerabilities and improve overall quality-of-life outcomes.

### 4.6 Limitations and future directions

This study's limitations include its single-center design and restricted sample size, although the repeated-measures approach enhances internal validity.

Future research should expand on the interaction between socioeconomic variables and postoperative QoL, examine differential effects across types of valve interventions, and compare structured care pathways with standard follow-up models. Additionally, qualitative approaches may enrich understanding of the subjective meaning of recovery from the patient's perspective.

## 5 Conclusion

This longitudinal study demonstrated that cardiac valve surgery is associated with significant improvements in patients' perceived quality of life, particularly in the domains of mobility, pain/discomfort, self-care, and emotional and social functioning, assessed at three perioperative time points. By incorporating validated patient-reported outcome measures (SF-36 and EQ-5D) within a structured Surgical Valve Disease Care Line, the findings indicate that longitudinal and multidisciplinary follow-up consistently contributes to functional and psychosocial recovery, reinforcing surgery as a central therapeutic strategy and highlighting the relevance of organized, patient-centered care models for sustained improvements in quality of life after surgical intervention.

## Data Availability

The raw data supporting the conclusions of this article will be made available by the authors, without undue reservation.
