# Supplementary material for: The perception of surgical valve disease patients on quality of life improvement through the care line model: a longitudinal study
Source: Front Cardiovasc Med. 2026 Jan 23;12:1489309. doi: 10.3389/fcvm.2025.1489309 (PMC12879752; doi:10.3389/fcvm.2025.1489309)
Supplement: Supplementary file 1 [file Table1.docx]

| **Regression Model** | **Social Functioning** | | **Functional Capacity** | | **Pain** | | **General Health** | | **Physical Aspects** | | **Role-Emotional** | | **Mental Health** | | **Vitality** | |
| --- | --- | --- | --- | --- | --- | --- | --- | --- | --- | --- | --- | --- | --- | --- | --- | --- |
|  | Normal | Poisson | Normal | Poisson | Normal | Poisson | Normal | Poisson | Normal | Poisson | Normal | Poisson | Normal | Poisson | Normal | Poisson |
| **Time** | 401075,98 | -164547,65 | 339164,76 | -119354,52 | 353107,62 | -148921,54 | 249587,9 | -169049,45 | 702210,42 | -168131,78 | 573492,71 | -104960,24 | 235335,61 | -172215,10 | 95667,51 | -154240,39 |
| **Sex** | 432987,98 | -164063,31 | 410024,47 | -118035,07 | 353662,70 | -148914,59 | 260530,46 | -168878,17 | 737146,74 | -167611,77 | 759856,62 | -101026,63 | 226330,36 | -172346,35 | 99776,94 | -154176,78 |
| **Age group** | 440262,84 | -163953,07 | 424880,36 | -117751,93 | 360801,80 | -148798,56 | 267500,52 | -168775,20 | 747347,93 | -167461,07 | 765971,66 | -100894,11 | 240629,88 | -172138,64 | 103299,59 | -154120,31 |
| **Education level** | 425609,64 | -161659,92 | 406038,91 | -116806,07 | 348479,92 | -146578,67 | 261911,82 | -166499,81 | 736147,72 | -164070,13 | 730454,89 | -99708,37 | 235413,88 | -169721,11 | 100711,85 | -151361,23 |
| **Professional activity** | 426444,34 | -164155,36 | 409537,29 | -118042,12 | 347856,91 | -149003,28 | 261834,48 | -168853,13 | 736810,68 | -167603,96 | 750640,99 | -101232,70 | 238356,14 | -172163,71 | 102773,50 | -154122,17 |
| **Smoking** | 419553,24 | -164303,54 | 423675,17 | -117774,52 | 352852,12 | -148935,52 | 268882,66 | -168752,26 | 723015,06 | -167871,45 | 761164,26 | -101005,82 | 227048,19 | -172352,25 | 98869,03 | -154191,88 |
| **Alcohol and drugs** | 440490,87 | -163949,18 | 424875,02 | -117752,67 | 357277,67 | -148861,42 | 269300,68 | -168748,69 | 750286,23 | -167416,91 | 764967,74 | -100915,74 | 236829,99 | -172198,87 | 101668,36 | -154146,35 |
| **Psychiatric and psychological history** | 427691,30 | -164157,38 | 419956,21 | -117853,31 | 348829,55 | -149007,94 | 268792,76 | -168755,76 | 730881,46 | -167731,49 | 751407,22 | -101246,52 | 233740,11 | -172243,68 | 100795,47 | -154161,33 |
| **Psychotropic medications** | 421040,54 | -164267,84 | 402467,27 | -118233,50 | 334714,57 | -149265,71 | 264889,82 | -168816,36 | 724123,48 | -167842,48 | 745044,04 | -101404,71 | 226562,10 | -172357,13 | 99449,55 | -154183,63 |
| **Etiology** | 439905,69 | -163953,50 | 414972,61 | -117931,08 | 349823,41 | -148975,57 | 263316,71 | -168832,13 | 734781,45 | -167659,53 | 748568,43 | -101271,93 | 237988,98 | -172172,99 | 102951,51 | -154120,11 |
| **Procedure performed** | 440026,52 | -163954,71 | 425122,32 | -117745,39 | 348865,75 | -148998,29 | 268106,58 | -168764,20 | 740991,07 | -167557,45 | 765012,29 | -100913,41 | 240163,30 | -172143,51 | 103206,91 | -154119,86 |
| **Type of valve** | 440385,99 | -163949,24 | 425422,71 | -117739,46 | 358586,02 | -148832,61 | 269327,12 | -168745,93 | 746166,63 | -167475,53 | 763254,86 | -100950,62 | 239723,15 | -172150,54 | 102657,15 | -154129,71 |
| **Multivariate** | 333658,94 | -163107,43 | 280212,05 | -119207,42 | 289401,54 | -149971,50 | 217902,79 | -169507,89 | 627404,47 | -169305,85 | 505293,62 | -104560,88 | 188098,26 | -172922,52 | 84179,71 | -154417,54 |

**Table 1 - QIC Values for Regression Models of the SF-36 Instrument Domains**
