# Supplementary material for: The perception of surgical valve disease patients on quality of life improvement through the care line model: a longitudinal study
Source: Front Cardiovasc Med. 2026 Jan 23;12:1489309. doi: 10.3389/fcvm.2025.1489309 (PMC12879752; doi:10.3389/fcvm.2025.1489309)
Supplement: Supplementary file 2 [file Datasheet1.pdf]

**SUPPLEMENTARY MATERIAL**

**SF-36 and EQ-5D Instruments**

**Name:_____________________________ Hospital Record (RGHC): __________**

## SF-36 – Health Survey

**Instructions:** This survey asks questions about your health. This information will help us understand how you feel and how well you are able to perform your daily activities. Please answer each question by marking the response as indicated. If you are unsure how to answer, please try to answer as best as you can.

**1. In general, would you say your health is: (circle one)**

Excellent ................................................. 1
Very good .................................................. 2
Good ....................................................... 3
Fair ......................................................... 4
Poor ........................................................ 5

**2. Compared to one year ago, how would you rate your health in general now? (circle one)**

Much better now than one year ago ............................ 1
Somewhat better now than one year ago ...................... 2
About the same as one year ago ................................... 3
Somewhat worse now than one year ago ..................... 4
Much worse now than one year ago .............................5

**3. The following items are about activities you might do during a typical day. Because of your health, do you have difficulty performing these activities? If so, how much?** (circle one number for each line)

| **Activities** | **Yes, limited a lot** | **Yes, limited a little** | **No, not limited at all** |
|---|---|---|---|
| **a. Vigorous activities (running, lifting heavy objects, strenuous sports)** | 1 | 2 | 3 |
| **b. Moderate activities (moving a table, vacuuming, bowling, sweeping)** | 1 | 2 | 3 |
| **c. Lifting or carrying groceries** | 1 | 2 | 3 |
| **d. Climbing several flights of stairs** | 1 | 2 | 3 |
| **e. Climbing one flight of stairs** | 1 | 2 | 3 |
| **f. Bending, kneeling, or stooping** | 1 | 2 | 3 |
| **g. Walking more than one kilometer** | 1 | 2 | 3 |
| **h. Walking several blocks** | 1 | 2 | 3 |
| **i. Walking one block** | 1 | 2 | 3 |
| **j. Bathing or dressing yourself** | 1 | 2 | 3 |

**4. During the past 4 weeks, have you had any of the following problems with your work or other regular daily activities as a result of your physical health?** (circle one for each line)

| | **Yes** | **No** |
|---|---|---|
| **a. Cut down the amount of time spent on work or activities** | 1 | 2 |
| **b. Accomplished less than you would like** | 1 | 2 |
| **c. Were limited in the kind of work or activities** | 1 | 2 |
| **d. Had difficulty performing work or activities (extra effort required)** | 1 | 2 |

**5. During the past 4 weeks, have you had any of the following problems with your work or other regular daily activities as a result of emotional problems (such as feeling depressed or anxious)?**

| | Yes | No |
|---|---|---|
| **a. Cut down the amount of time spent on work or activities** | 1 | 2 |
| **b. Accomplished less than you would like** | 1 | 2 |
| **c. Did not do work or activities as carefully as usual** | 1 | 2 |

**6. During the past 4 weeks, to what extent has your physical health or emotional problems interfered with your normal social activities (family, friends, neighbors, or groups)?** (circle one)

Not at all ............. 1
Slightly ................. 2
Moderately ........... 3
Quite a bit ............ 4
Extremely ............ 5

**7. How much bodily pain have you had during the past 4 weeks?**

(circle one)

None ................. 1
Very mild .......... 2
Mild .................. 3
Moderate .......... 4
Severe .............. 5
Very severe ...... 6

**8. During the past 4 weeks, how much did pain interfere with your normal work (including work outside the home and housework)?**

(circle one)

Not at all ............ 1
A little ................. 2
Moderately .......... 3
Quite a bit ........... 4
Extremely ........... 5

**9. These questions are about how you feel and how things have been during the past 4 weeks. For each question, please give the answer that best describes how you have felt.** (circle one number for each line)

| | **All of the time** | **Most of the time** | **A good bit of the time** | **Some of the time** | **A little of the time** | **None of the time** |
|---|---|---|---|---|---|---|
| **a. Felt full of life** | 1 | 2 | 3 | 4 | 5 | 6 |
| **b. Felt very nervous** | 1 | 2 | 3 | 4 | 5 | 6 |
| **c. Felt so down nothing could cheer you up** | 1 | 2 | 3 | 4 | 5 | 6 |
| **d. Felt calm and peaceful** | 1 | 2 | 3 | 4 | 5 | 6 |
| **e. Felt energetic** | 1 | 2 | 3 | 4 | 5 | 6 |
| **f. Felt downhearted and depressed** | 1 | 2 | 3 | 4 | 5 | 6 |
| **g. Felt worn out** | 1 | 2 | 3 | 4 | 5 | 6 |
| **h. Felt happy** | 1 | 2 | 3 | 4 | 5 | 6 |
| **i. Felt tired** | 1 | 2 | 3 | 4 | 5 | 6 |

**10. During the past 4 weeks, how much of the time has your physical health or emotional problems interfered with your social activities (visiting friends, relatives, etc.)?**

All of the time ........... 1
Most of the time ........ 2
Some of the time ........ 3
A little of the time ...... 4
None of the time ......... 5

**11. How true or false is each of the following statements for you?**

| | **Definitely true** | **Mostly true** | **Not sure** | **Mostly false** | **Definitely false** |
|---|---|---|---|---|---|
| **a. I seem to get sick a little easier than other people** | 1 | 2 | 3 | 4 | 5 |

| | | | | | |
|---|---|---|---|---|---|
| **b. I am as healthy as anyone I know** | 1 | 2 | 3 | 4 | 5 |
| **c. I expect my health to get worse** | 1 | 2 | 3 | 4 | 5 |
| **d. My health is excellent** | 1 | 2 | 3 | 4 | 5 |

*Developed by RAND Corporation © as part of the Medical Outcomes Study*

# EQ - 5D

**Health Questionnaire (Brazilian Version)**

**EQ-5D – Health Questionnaire (Brazilian Version)**

Please tick (✓) **one box** in each of the following groups to indicate which statement best describes your health **today**.

**Mobility**

- I have no problems walking ☐
- I have some problems walking ☐
- I am confined to bed ☐

**Self-Care**

- I have no problems with self-care ☐
- I have some problems washing or dressing myself ☐
- I am unable to wash or dress myself ☐

**Usual Activities** (work, study, housework, family or leisure activities)

- I have no problems performing my usual activities ☐
- I have some problems performing my usual activities ☐
- I am unable to perform my usual activities ☐

**Pain / Discomfort**

- I have no pain or discomfort ☐
- I have moderate pain or discomfort ☐
- I have extreme pain or discomfort ☐

**Anxiety / Depression**

- I am not anxious or depressed ☐
- I am moderately anxious or depressed ☐
- I am extremely anxious or depressed ☐

The best imaginable health state

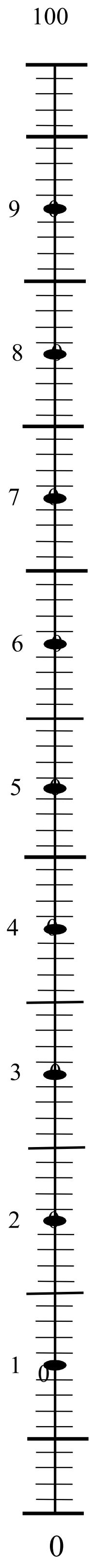


The worst imaginable health

To help people describe how good or bad their health state is, we have drawn a scale (similar to a thermometer), in which the best imaginable health state is marked as 100 and the worst imaginable health state is marked as 0.

We would like you to indicate on this scale how good or bad your health state is today, in your opinion. Please draw a line from the box below to the point on the scale that best represents your health state today.

**Your health today**

**Sociodemographic Information**

Because all responses are anonymous, we will be better able to understand the answers if we have some of your personal information, as covered in the following questions.

1. Have you had contact with serious illness?

- Yourself ☐ Yes ☐ No
- In your family ☐ Yes ☐ No
- While caring for others ☐ Yes ☐ No

2. Age: ______ years

3. Sex: ☐ Male ☐ Female

4. Smoking status:
   ☐ Smoker
   ☐ Former smoker
   ☐ Never smoked

5. Have you ever worked in health or social services? ☐ Yes ☐ No
   If yes, specify role: ______________________________

6. Current occupation:
   ☐ Employed / Self-employed
   ☐ Retired
   ☐ Homemaker
   ☐ Student
   ☐ Seeking employment
   ☐ Other (specify): ____________________________

7. Did you continue education beyond compulsory schooling? ☐ Yes ☐ No

8. Do you have a university degree or equivalent qualification? ☐ Yes ☐ No

9. Postal code (if known): _______________________
